# Cryoballoon ablation of atrial fibrillation in octogenarians: one year outcomes from the cryo global registry

**DOI:** 10.1007/s10840-023-01680-z

**Published:** 2023-12-12

**Authors:** Dennis Lawin, Thorsten Lawrenz, K. R. Julian Chun, Hong-Euy Lim, Valentine Obidigbo, Jada M. Selma, Peter Peytchev, Dinh Quang Nguyen, Csaba Földesi, Christoph Stellbrink, the Cryo AF Global Registry Investigators

**Affiliations:** 1 Department of Cardiology and Intensive Care Medicine, University Hospital OWL of Bielefeld University, Campus Klinikum Bielefeld, 50 Teutoburger Street Bielefeld, 33604 Bielefeld, Germany; 2 Cardioangiologisches Centrum Bethanien, Frankfurt, Germany; 3 Hallym University Sacred Heart Hospital, Anyang, Republic of Korea; 4 Medtronic, Inc., Minneapolis, MN USA; 5 Onze-Lieve-Vrouwziekenhuis, Aalst, Belgium; 6 St. Vinzenz-Hospital Köln, Cologne, Germany; 7 Gottsegen György Országos Kardiovaszkuláris Intézet, Budapest, Hungary

**Keywords:** Atrial fibrillation, Catheter ablation, Cryoballoon, Octogenarians, Registry

## Abstract

**Background:**

Limited information is available on the safety and efficacy of cryoballoon ablation (CBA) in elderly patients with atrial fibrillation (AF). Moreover, global utilization of CBA in this population (≥ 80 years old) has not been reported. This study’s objectives were to determine the use, efficacy, and safety of CBA to treat octogenarians suffering from AF.

**Methods:**

In this sub-analysis of the Cryo Global Registry, 12-month outcomes of treating AF via CBA in octogenarians were compared to patients < 80 years old. Efficacy was evaluated as time to a ≥ 30 s atrial arrhythmia (AA) recurrence. Healthcare utilization was determined via repeat ablations and hospitalizations. Improvement upon disease burden was evaluated through patient reporting of symptoms and the EQ-5D-3L quality of life (QoL) survey.

**Results:**

The octogenarian cohort (n = 101) had a higher prevalence of females (51.5% vs 35.7%) and CHA_2_DS_2_-VASc scores (4.2 ± 1.3 vs 2.0 ± 1.5) compared to the control cohort (n = 1573, both p < 0.01). Even when adjusting for baseline characteristics and antiarrhythmic drug usage, freedom from AA recurrence at 12 months (80.6% vs 78.9%, HR_adj_:0.97 [95% CI:0.59–1.58], p = 0.90) was comparable between octogenarians and control, respectively. Similar serious adverse event rates were observed between octogenarians (5.0%) and control (3.2%, p = 0.38). The groups did not differ in healthcare utilization nor reduction of AF-related symptoms from baseline to follow-up, but both experienced an improvement in QoL at 12 months.

**Conclusions:**

Despite more age-related comorbidities, CBA is a safe and effective treatment for AF in octogenarians, with efficacy and adverse events rates akin to ablations performed in younger patients.

**Clinical trial registration:**

https://clinicaltrials.gov/ct2/show/NCT02752737

**Graphical Abstract:**

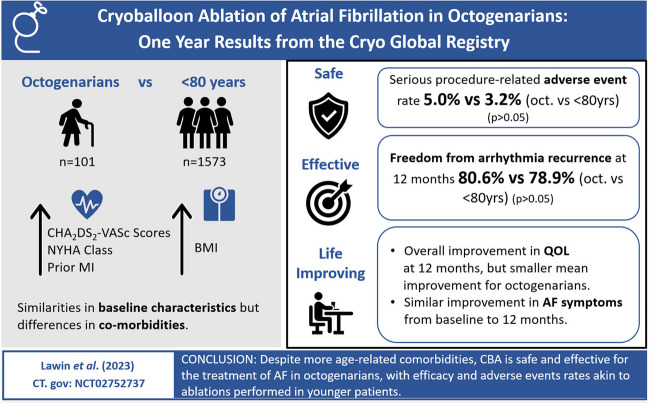

**Supplementary Information:**

The online version contains supplementary material available at 10.1007/s10840-023-01680-z.

## Introduction

In 2017, the approximate global prevalence of atrial fibrillation (AF) was 37.6 million, and it was forecasted to increase by more than 60% by 2050 [1]. A growing elder population has contributed to this escalation of AF prevalence. Presently, AF impacts 10–17% of people 80 years and older [2]. Managing AF in elderly patients can be difficult due to numerous comorbidities [2]. Therefore, some physicians are hesitant to perform minimally invasive procedures, such as cryoballoon ablation (CBA), in this population [3]. Furthermore, large clinical trials evaluating the safety and efficacy of CBA typically have not included octogenarians, even though they comprise a substantial proportion of the AF population [4–7].

Smaller studies have demonstrated the safety and efficacy of CBA in patients ≥ 75 years old with AF [3, 6, 8–10]. However, these studies involved one or multiple centers from the same country, and thus lacked diversity in patient demographics, standard of care protocols, and physician experience [3, 6, 8–10]. To address these evidence gaps, this sub-study from the *Cryo AF Global Registry* reports real-world efficacy, safety, healthcare utilization, and quality of life (QoL) outcomes in octogenarian AF patients treated via CBA.

## Methods

### Study Design

The *Cryo Global Registry* (NCT02752737) is an ongoing, prospective, multicenter, post-market, Medtronic-sponsored registry which evaluates AF ablation using the Arctic Front family of cryoablation catheters (Medtronic, Inc). An international physician steering committee supervised data quality and collection, analyses, and publication timelines. The principles defined in the Declaration of Helsinki and Good Clinical Practice guidelines drove data collection methods. Each site obtained authorization from local institutional/ethics review boards and acquired consent from patients preceding study enrollment.

### Patient Population

All patients were required to be ≥ 18 years old with a planned CBA procedure and were not disqualified for any preexisting characteristics nor medical conditions. In this examination, patients with paroxysmal or persistent AF enrolled between May 2016 and May 2020 at global centers who treated at least one patient with an age ≥ 80 years were included. This sub-analysis compares two cohorts: patients ≥ 80 years of age (octogenarian group) and patients < 80 years of age (control group) at the time of enrollment.

### Cryoballoon Ablation Procedure

At each site, operators conducted the CBA procedure according to local standard-of-care and current guidelines. Briefly, transeptal puncture was used to gain access to the left atrium (LA). To deliver a 23- or 28-mm CBA catheter (Arctic Front Advance; Medtronic, Inc.) into the LA, a specialized 15-F outer diameter steerable sheath was employed (FlexCath Advance Steerable Sheath; Medtronic, Inc.). A J-tip guidewire or a dedicated inner-lumen octopolar/decapolar circular mapping catheter (Achieve or Achieve Advance; Medtronic, Inc.) was used to deliver the cryoballoon catheter and sheath to the desired pulmonary vein (PV). The cryoballoon was inflated and advanced toward the PV before each ablation. Freezing was initiated after antral occlusion of the targeted PV was validated by selective angiography. Operators dictated the number and length of cyroapplications per PV, and pulmonary vein isolation (PVI) was verified by entrance and/or exit block.

During right-sided CBA, pacing the diaphragm with a diagnostic catheter inserted into the right subclavian vein (or superior vena cava) and monitoring nerve function with manual and/or adjunctive diaphragmatic movement detection was recommended. All freezes were halted upon detection of a weakened diaphragmatic response. Operators independently decided on sedation method, pre- and intra-procedural imaging, esophageal temperature monitoring, chemical assessment of acute PVI, and placement of adjunctive ablation lesions. Patients were prescribed periprocedural anticoagulation and anti-arrhythmic drugs (AADs) and were discharged according to local standard-of-care procedures.

### Patient Follow-up and Study Endpoints

Patients were monitored post ablation according to each center’s standard-of-care, which included a mandatory 12-month post-procedure visit. Following a 90-day blanking period, efficacy was evaluated by the 12-month freedom from ≥ 30 s of documented AF/atrial flutter (AFL)/atrial tachycardia (AT). The rate of serious procedure-related adverse events was the primary safety endpoint. Serious events were defined as events that resulted in death or a serious deterioration in health.

Secondary endpoints included procedural characteristics, change in AF-related symptoms, QoL, and freedom from repeat ablation and rehospitalizations at 12 months. At baseline and 12-month follow-up, predefined AF-related symptoms and QoL were assessed. The European Quality of Life-5 Dimensions-3 Levels (EQ-5D-3L) questionnaire was utilized to evaluate QoL. All-cause and cardiovascular (CV)-related hospitalization and repeat ablation rates were analyzed throughout 12 months of follow-up. Class I or III AAD prescription was collected at discharge and 12 months post-ablation.

### Statistical Analysis

Continuous variables were summarized as mean and standard deviation, and categorical variables were summarized as counts and percentages. Kaplan–Meier methods were used to estimate 12-month freedom from atrial arrhythmia (AA) recurrence, repeat ablation, and rehospitalization. Standard error was approximated with Greenwood’s formula. Cox regression models were employed to compute hazard ratios for efficacy outcomes between cohorts. Propensity score methods were utilized to evaluate an adjusted hazard ratio to account for dissimilarities in baseline characteristics between the octogenarian and control groups. With the classification of octogenarian serving as the dependent variable, a logistic regression model that encompasses all baseline characteristics (excluding CHA_2_DS_2_-VASc scores and age) as covariates was developed to calculate propensity scores. AAD usage following ablation was included as a covariate as well. To approximate adjusted hazard ratios, the Cox regression model comprised propensity score as a covariate. Distinct Cox regression models were utilized for each efficacy and healthcare utilization endpoint. Changes in number of symptoms and QoL from baseline to 12 months were assessed with linear models accounting for differences at baseline. Post-ablation AAD prescription at discharge (between octogenarians and control groups) was assessed with a Chi-Square test of independence, while change in the post-ablation AAD prescription from discharge to 12 months was assessed with a logistic model accounting for differences at discharge. P-values of < 0.05 were considered statistically significant.

## Results

### Patient and Baseline Characteristics

Of the 4210 patients enrolled and treated with CBA in the Cryo Global Registry by October 2021,1674 patients with paroxysmal AF (PAF) or persistent AF (PsAF) were treated in 14 countries at 37 global centers who ablated at least one octogenarian (Supplemental Table 1). Analysis cohorts were specified as patients ≥ 80 (octogenarian; n = 101) and patients < 80 years old (control; n = 1573). Baseline characteristics of the study population are displayed in Table 1. Average age of the octogenarians was 82 ± 2 vs 62 ± 10 years in the control group. Percentage of females was higher in the octogenarian population (51.5% vs 35.7%, p < 0.01). The octogenarian cohort had lower body mass index (26 ± 4 kg/m^2^ vs 27 ± 5 kg/m^2^), were more hypertensive (79.2% vs 55.5%), had higher CHA_2_DS_2_-VASc scores (4.2 ± 1.3 vs 2.0 ± 1.5), and had more severe heart failure-related symptoms according to NYHA class (all p < 0.01). There were no differences between the octogenarian and control cohorts in the proportion of patients with PAF (70.3% vs 74.4%), PsAF (24.8% vs 19.6%), or long-standing persistent (5.0% vs 6.0%) AF (all p > 0.05), respectively. Octogenarian and control cohorts were similar with regards to years diagnosed with AF (2.9 ± 4.9 vs 3.2 ± 4.8), left ventricular ejection fraction (58 ± 12% vs 60 ± 9%), and left atrial diameter (43 ± 8 vs 42 ± 8 mm, all p > 0.05). Both groups failed a similar number of AADs at baseline (0.6 ± 0.8 vs 0.7 ± 0.7, p = 0.19).

**Table 1:** Baseline Patient Characteristics (Octogenarians vs. Control)

Subject Characteristics	Octogenarians (N = 101)	Control (N = 1573)	p-value ^*^
Female sex (N (%))	52 (51.5%)	562 (35.7%)	< 0.01
Age in years (mean ± SD)	82 ± 2	62 ± 10	< 0.01
Body mass index in kg/m2 (mean ± SD)	26 ± 4	27 ± 5	< 0.01
CHA_2_DS_2_-VASc score (mean ± SD) ^†^	4.2 ± 1.3	2.0 ± 1.5	< 0.01
Type of AF (N (%))			0.46
*Paroxysmal AF*	71 (70.3%)	1170 (74.4%)	
*Persistent (0–12 months)*	25 (24.8%)	308 (19.6%)	
*Long standing persistent (*> *12 months)*	5 (5.0%)	95 (6.0%)	
Years diagnosed with AF (mean ± SD) ^‡^	2.9 ± 4.9	3.2 ± 4.8	0.56
History of atrial flutter (N (%))	12 (11.9%)	158 (10.0%)	0.5
History of atrial tachycardia (N (%))	2 (2.0%)	33 (2.1%)	> 0.99
Left atrial diameter in mm (mean ± SD) ^§^	43 ± 8	42 ± 8	0.46
Left ventricular ejection fraction % (mean ± SD) ^¶^	58 ± 12	60 ± 9	0.09
Number of previously failed AADs (mean ± SD) ^#^	0.6 ± 0.8	0.7 ± 0.7	0.19
Baseline NYHA (N (%)) ^Ω^			< 0.01
*Class I*	8 (7.9%)	192 (12.2%)	
*Class II*	20 (19.8%)	128 (8.1%)	
*Class III*	10 (9.9%)	56 (3.6%)	
*Class IV*	0 (0.0%)	1 (0.1%)	
Hypertension	80 (79.2%)	873 (55.5%)	< 0.01
Prior cardiac device implant	10 (9.9%)	73 (4.6%)	0.03
Prior myocardial infarction (N (%))	7 (6.9%)	40 (2.5%)	0.02
Prior stroke/transient ischemic attack (N (%))	10 (9.9%)	99 (6.3%)	0.15
History of vascular diseases	10 (9.9%)	57 (3.6%)	< 0.01
History of thromboembolism	4 (4.0%)	25 (1.6%)	0.09
History of coronary artery disease (N (%))	18 (17.8%)	188 (12.0%)	0.09
Diabetes (N (%))	17 (16.8%)	200 (12.7%)	0.22
Sleep apnea (N (%))	7 (6.9%)	68 (4.3%)	0.21

Abbreviations; AF, atrial fibrillation; AAD, antiarrhythmic drug; NYHA, New York Heart Association; SD, standard deviation

^†^ 1531 patients with CHA_2_DS_2_-VASc score reported; 88 octogenarians, 1443 control

^‡^ 1570 patients with AF diagnosis date reported; 90 octogenarians, 1480 control

^§^ 1064 patients with left atrial diameter reported; 61 octogenarians, 1003 control

^¶^ 1369 patients with left ventricular ejection fraction reported; 80 octogenarians, 1289 control

^#^ 1641 patients with number of failed AADs reported; 101 octogenarians, 1540 control

^Ω^ 143 patients not reported wit NYHA status; 13 octogenarians, 130 control

^*^ Statistical tests comparing octogenarians versus control cohort. Continuous variables compared with t-test; binary variables compared with exact test

### Procedural Characteristic

Table 2 displays procedural characteristics. The majority of patients underwent CBA using a 28-mm Arctic Front Advance (98.7%). Total procedure, LA dwell, and fluoroscopy times did not differ between groups (p > 0.05). On average, octogenarians were administered 1.5 ± 0.8 freezes per PV for a mean duration of 184 ± 52 secs in comparison to the control group who received 1.6 ± 1.0 freezes per PV for a mean duration of 177 ± 52 secs (p = 0.02 for applications and p < 0.01 for duration). Overall acute PVI was high (95.0% in the octogenarian vs. 96.2% in the control, p = 0.59). Following CBA, the number of days until discharge were higher in the octogenarian patients (2.6 ± 4.1 days) compared to controls (1.8 ± 2.8 days, p < 0.01), and 2.0% of octogenarians vs 8.9% of control patients were discharged the same day of the index procedure.

**Table 2:** Procedural Characteristics (Octogenarians vs. Control)

Procedural Characteristics	Octogenarians (N = 101)	Control (N = 1573)	p-value^*^
Total lab occupancy time in min (mean ± SD)^†^	136 ± 52	132 ± 53	0.42
Total procedure time in min (mean ± SD)^‡^	78 ± 29	76 ± 28	0.36
Left atrial dwell time in min (mean ± SD)^§^	55 ± 23	52 ± 22	0.23
Total cryo fluoroscopy time in min (mean ± SD)^¶^	17 ± 18	16 ± 22	0.46
Freeze duration per vein in secs (mean ± SD)	184 ± 52	177 ± 52	< 0.01
Number of applications per vein (mean ± SD)	1.5 ± 0.8	1.6 ± 1.0	0.02
Cryoballoon nadir temperature in °C (mean ± SD)	-48 ± 7	-48 ± 7	0.68
Sedation method (N (%))			0.25
*Conscious sedation*	66 (65.3%)	934 (59.4%)	
*General anesthesia*	35 (34.7%)	636 (40.4%)	
Esophageal temperature monitoring (N (%))	63 (62.4%)	937 (59.6%)	0.60
Phrenic nerve monitoring (not done) (N (%))	1 (1.0%)	8 (0.5)	0.43
*Pacing/palpate (N (*%))	94 (93.1%)	1403 (89.2%)	0.31
*Diaphragm stimulation (N (%))*	34 (33.7%)	473 (30.1%)	0.44
Pre-procedural imaging (CT and/or MRI) (N (%))	27 (26.7%)	437 (27.8%)	0.91
Pulmonary vein ablation acute success (N (%))^#^	96 (95.0%)	1514 (96.2%)	0.59
*PVI touch-up with focal cryo catheter (N (%))*	0 (0.0%)	1 (0.1%)	> 0.99
*PVI touch-up with focal RF catheter (N (%))*	4 (4.0%)	121 (7.7%)	0.24
Additional ablation lesion (N (%))			
*CTI line*	12 (11.9%)	200 (12.7%)	> 0.99
*Other non-PVI ablation*	3 (3.0%)	84 (5.3%)	0.48
Left atrium AF trigger	0 (0.0%)	17 (1.1%)	
Right atrium AF trigger	2 (2.0%)	5 (0.3%)	
Superior vena cava vein trigger	0 (0.0%)	12 (0.8%)	
Inferior vena cava vein trigger	0 (0.0%)	0 (0.0%)	
Mitral valve isthmus or line	0 (0.0%)	5 (0.3%)	
Left sided roofline	2 (2.0%)	17 (1.1%)	
CFAE	0 (0.0%)	6 (0.4%)	
Other	2 (2.0%)	31 (2.0%)	
Length of stay for cryoballoon procedure in days	2.6 ± 4.1	1.8 ± 2.8	< 0.01^1^
*Median [IQR]*	2 [1,2]	2 [1,2]	
*Same day discharge (0 day stay)* (N (%))	2 (2.0%)	141 (8.9%)	
*1 day (1 overnight)* (N (%))	39 (38.6%)	835 (53.2%)	
*2 days (2 overnight)* (N (%))	38 (37.6%)	382 (24.3%)	
*3 days (3 overnight)* (N (%))	8 (7.9%)	96 (6.1%)	

Abbreviations: CFAE, complex fractionated atrial electrograms; CTI, cavo-tricuspid isthmus; CT, computerized tomography; ICE, intracardiac echocardiography; MRI, magnetic resonance imaging; PVI, pulmonary vein isolation; RF, radiofrequency

^†^ 1663 patients reported lab occupancy time; 100 octogenarians, 1563 control

^‡^ 1664 patients reported procedure time; 101 octogenarians, 1563 control

^§^ 1666 patients reported LA dwell time; 101 octogenarians, 1565 control

^¶^ 1562 patients reported cryo fluoroscopy time; 88 octogenarians, 1474 control

^#^ All targeted pulmonary veins isolated after cryoballoon ablation and focal touch-up

* t-test for continuous variables, exact test for binary variables

^1^ Wilcoxon rank-sum test

### Patient Follow-up

**Fig. 1:**
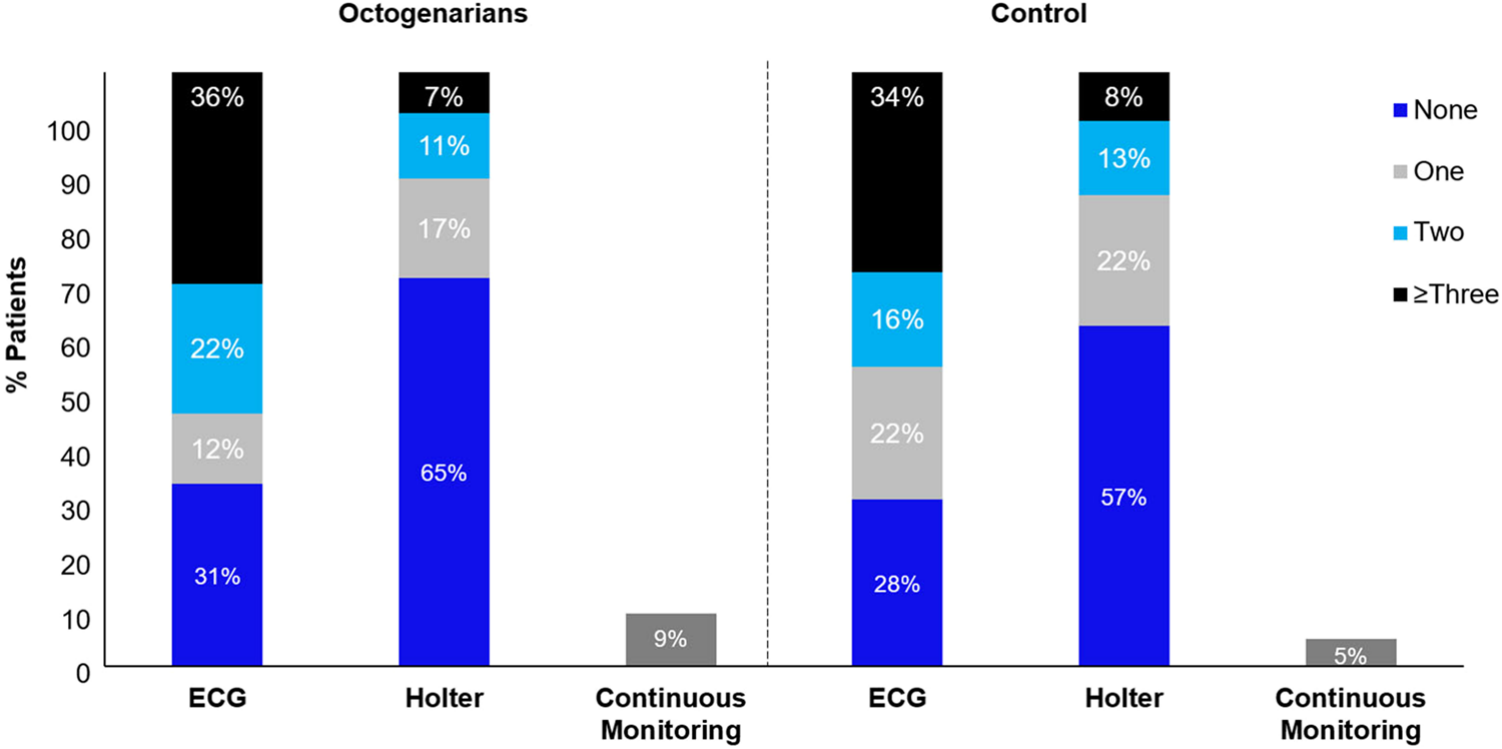
Rhythm Monitoring during 12-Month Follow-up. Percentage of patients monitored for atrial arrhythmia recurrences with none (blue), one (gray), two (aqua) or three or more (black) 12-lead ECGs or Holter monitoring events over the 12-month follow-up period. Proportion of patients with continuous monitoring (pacemaker/implantable cardiac monitor) are shown in dark gray

Before the 12-month visit, eight patients from the octogenarian group (7.9%) and 63 patients from the control (4.0%) exited the study, while one (1.0%) octogenarian patient and 12 control (0.8%) patients died (p = 0.80, [95% CI: -0.018 – 0.022]; all unrelated to the CBA procedure). Rhythm monitoring was performed at least once during 12 months in 89 (88.1%) octogenarians and 1465 (93.1%) control patients (p = 0.07). The proportion of patients that were not monitored at all over 12 months did not differ between the cohorts (p = 0.06). During follow-up, Holter and/or ECG monitoring was conducted in 35 (34.7%) and 70 (69.3%) octogenarians compared to 670 (42.6%) and 1130 (71.8%) control patients, respectively. As displayed in Fig. 1, the cohorts experienced comparable type and frequency of rhythm monitoring.

### Efficacy and Safety

**Fig. 2:**
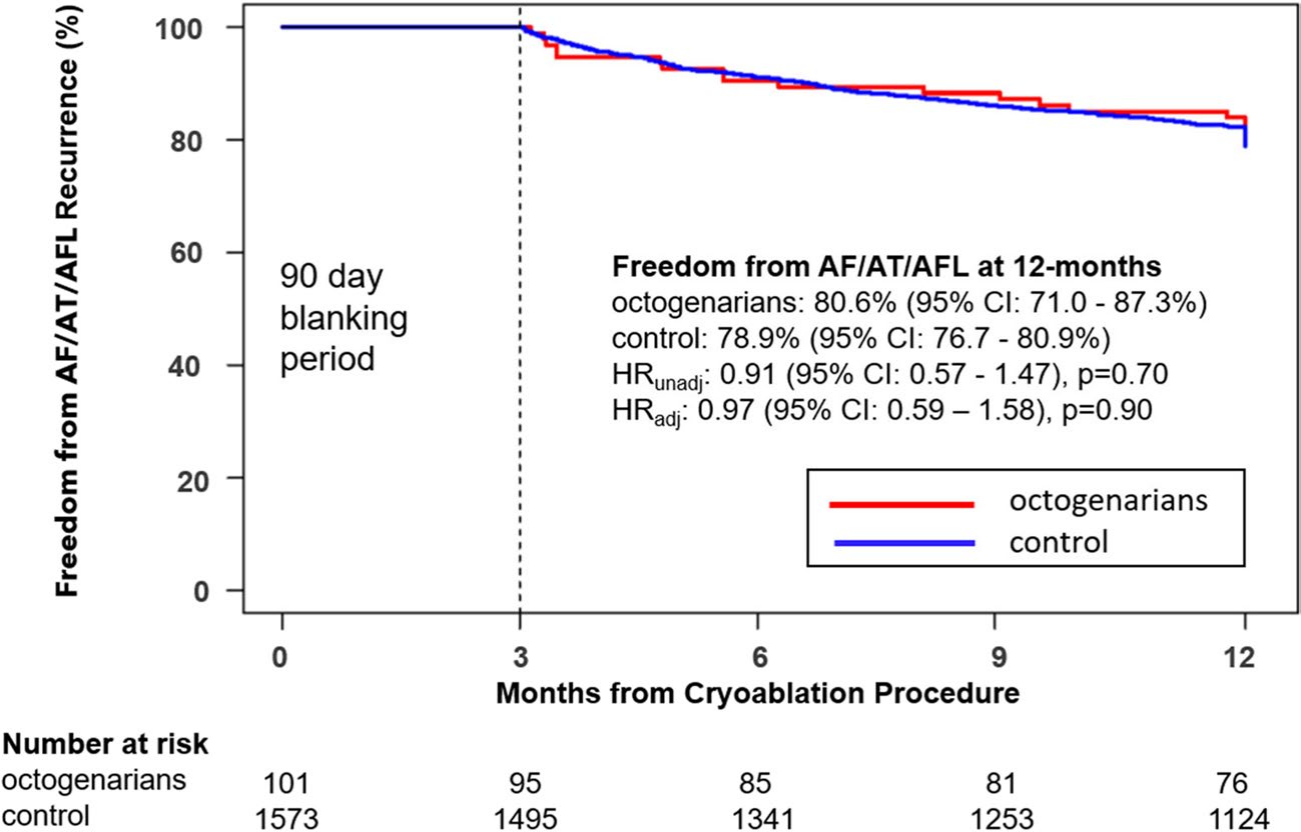
Freedom from Atrial Arrhythmia Recurrence at 12 Months. Kaplan–Meier estimate of freedom from ≥ 30 s recurrences of AF/AFL/AT at 12 months after a 90-day blanking period in octogenarians (red line) or controls (blue line) treated with CBA. Hazard ratios are presented from unadjusted (HR_unadj_) and adjusted (HR_adj_) Cox regression models, where the adjusted model include all subject characteristics from Table 1 with the exception of CHA_2_DS_2_-VASc score and age

Freedom from AF/AFL/AT recurrence at 12 months was not different between the octogenarian (80.6%, 95% CI: 71.0 – 87.3%) and control group (78.9%, 95% CI: 76.7 – 80.9%, HR_unadj_: 0.91 [95% CI: 0.57–1.47], p = 0.70; Fig. 2). Even accounting for differences in baseline characteristics and post-ablation AAD usage with propensity score methods, the hazard ratio for octogenarians vs control was not significant (HR_adj_: 0.97 [95% CI: 0.59–1.58], p = 0.90 Fig. 2). A total of 63 serious procedure-related adverse events were reported in 55 patients. No differences were observed between octogenarian (5.0%) and control patients (3.2%) with respect to serious adverse events (p = 0.38, Table 3). Of note, there was no reported cardiac tamponade, perforation, pericardial effusion, phrenic nerve paralysis, stroke, atrioesophageal fistula, or PV stenosis related to the procedure in the octogenarian cohort. ​Table 3 imparts a complete list of serious procedure-related adverse events.

**Table 3:** Serious Procedure-related Adverse Events (Octogenarians vs. Control)

	Number of Events (Number, % Patients)		
Serious Procedure-Related Complications	Octogenarians (N = 101)	Control (N = 1573)	p-value^*^
Total	**5 (5, 5.0)**	**58 (50, 3.2)**	**0.38**
Atrial septal defect	0 (0,0.0)	1 (1, 0.1)	
Cardiac failure	0 (0, 0.0)	2 (2, 0.1)	
Cardiac tamponade, perforation, or pericardial effusion	0 (0, 0.0)	5 (5, 0.3)	
Femoral artery aneurysm	0 (0, 0.0)	1 (1, 0.1)	
Groin-site complication^†^	1 (1, 1.0)	15 (13, 0.8)	
Headache	0 (0, 0.0)	1 (1, 0.1)	
Lip injury	0 (0, 0.0)	1 (1, 0.1)	
Myocardial infarction or ischemic cardiac event^‡^	0 (0, 0.0)	1 (1, 0.1)	
Pericarditis	0 (0, 0.0)	1 (1, 0.1)	
Phrenic nerve paralysis	0 (0, 0.0)	7 (7, 0.4)	
Postoperative hypotension	0 (0, 0.0)	3 (3, 0.2)	
Pulmonary or bronchial complication^§^	2 (2, 2.0)	6 (6, 0.4)	
Pyrexia	0 (0, 0.0)	2 (2, 0.1)	
Sepsis	1 (1, 1.0)	0 (0, 0.0)	
Stress cardiomyopathy	0 (0, 0.0)	0 (0, 0.0)	
Stroke or transient ischemic attack^¶^	0 (0, 0.0)	2 (2, 0.1)	
Supraventricular arrhythmia recurrences^#^	0 (0, 0.0)	7 (7, 0.4)	
Urinary retention	1 (1, 1.0)	1 (1, 0.1)	

^†^Hematoma, vascular pseudoaneurysm (ruptured), vessel puncture site discharge/ hematoma, arteriovenous fistula, femoral artery dissection, incision/puncture site hematoma, vascular access site hemorrhage

^‡^Angina pectoris, coronary arteriospasm, myocardial infarction

^§^Hematemesis, hemoptysis, hypercapnia, pneumothorax, pulmonary embolism, pneumonia, pleurisy

^¶^Cerebral infarction, cerebrovascular accident, ischemic stroke, lacunar stroke

^#^Atrial fibrillation, atrial flutter, atrial tachycardia, nodal arrhythmia, sinus bradycardia, supraventricular tachycardia with onset prior to index procedure discharge

^*^Exact test

### Healthcare Utilization

**Fig. 3:**
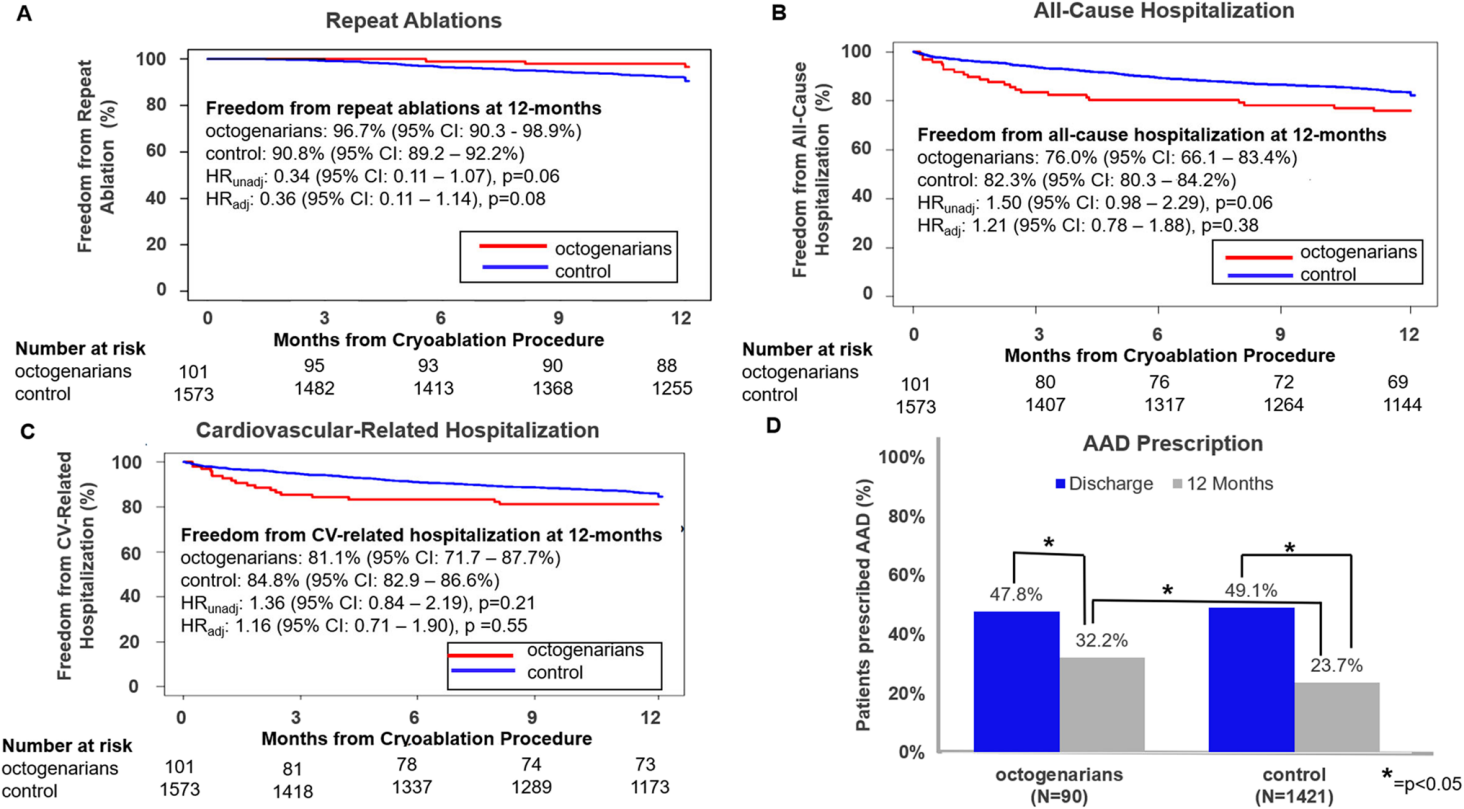
Freedom from Repeat Ablations, Hospitalization, and Antiarrhythmic Drug Usage. Kaplan–Meier estimate of (**A**) freedom from repeat ablation at 12 months, (**B**) freedom from all-cause hospitalization at 12 months, and (**C**) freedom from CV-related hospitalization at 12 months in octogenarians (red lines) or controls (blue lines) treated with CBA. Hazard ratios are presented from unadjusted (HR_unadj_) and adjusted (HR_adj_) Cox regression models, where the adjusted model include all subject characteristics from Table 1 with the exception of CHA_2_DS_2_-VASc score and age. (**D**) AAD prescription in octogenarians and controls at discharge (blue) and at 12 months (gray)

Octogenarians were not more likely to receive a repeat ablation (3.3%, 95% CI: 1.1 – 9.7%) in comparison to the control group (9.2%, 95% CI: 7.8 – 10.8%, HR_unadj_: 0.34 [95% CI: 0.11 – 1.07], p = 0.06, HR_adj_: 0.36 [95% CI: 0.11 – 1.14], p = 0.08); Fig. 3A). All-cause hospitalization was not statistically different between cohorts (HR_unadj_: 1.50 [95% CI: 0.98 – 2.29], p = 0.06, HR_adj_: 1.21 [95% CI: 0.78 – 1.88], p = 0.38), with 76.0% in the octogenarian cohort (95% CI: 66.1 – 83.4%) and 82.3% in the control cohort (95% CI: 80.3 – 84.2%) being free of an all-cause hospitalization during follow-up (Fig. 3B). However, octogenarians spent more days in the hospital during their first all-cause hospitalization post-ablation (8.3 ± 12.8 vs 4.3 ± 6.3 days, p = 0.014). The estimate for freedom from cardiovascular-related hospitalization was 81.1% (95% CI: 71.7–87.7%) in octogenarians and 84.8% (95% CI: 82.9–86.6%) in the control (HR_unadj_: 1.36 [95% CI: 0.84 – 2.19], p = 0.21, HR_adj_: 1.16 [95% CI: 0.71 – 1.90], p = 0.55; Fig. 3C). When comparing octogenarians to the control, AF was the predominant cause for all-cause (30.4% vs 55.2%, p = 0.073) and CV-related hospitalizations (38.9% vs 66.4%, p = 0.065) in both cohorts, respectively. AAD usage was significantly reduced for both groups from discharge to the 12-month follow-up (Fig. 3D). The post-ablation AAD prescription rate was similar in both groups at discharge (47.8% vs 49.1%, p = 0.80) but was significantly higher in the octogenarians at 12 months (32.2% vs 23.7%, p = 0.04) in comparison to the control (Fig. 3D), respectively.

### Quality of Life and Arrhythmia Symptoms

**Fig. 4:**
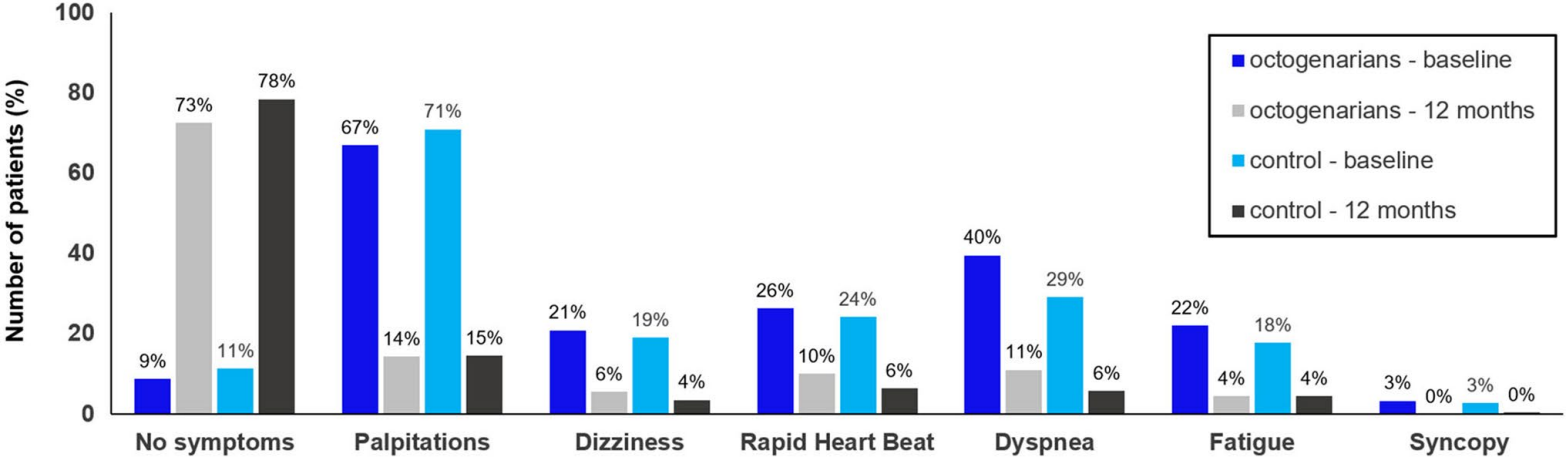
Atrial Fibrillation-Related Symptoms. Distribution of AF-related symptoms in octogenarians at baseline (blue) and 12 months (gray) and in controls at baseline (turquoise) and 12 months (black). A total of 1512/1674 patients reported symptoms

The distribution of each AF-related symptom at baseline and 12-month visit is shown in Fig. 4. At baseline, palpitations were the most prevalent symptom, present in 67.0% of the octogenarians and 70.9% of the control group. Despite poorer cardiovascular health, there was no significant difference in symptom burden for octogenarians in comparison to controls at baseline (p = 0.23). Twelve months post ablation, 72.5% of the octogenarians and 78.4% of the control group indicated no symptoms. There was no difference between groups in the reduction of AF-related symptoms from baseline to follow-up (p = 0.37). Octogenarians suffered from a significantly lower overall QoL at baseline (0.83 vs 0.89, p < 0.01) and experienced a smaller mean improvement in QoL at 12 months after CBA (0.020 vs 0.033, p < 0.01; Table 4).

**Table 4:** Quality of Life

EQ5D (N = 1428^†^)	Octogenarians (N = 83)	Control (N = 1345)	p-value
Baseline	0.83 ± 0.16	0.89 ± 0.13	< 0.01^†^
12-months	0.85 ± 0.15^§^	0.93 ± 0.12^¶^	< 0.01^‡^
Absolute difference	0.020 ± 0.186	0.033 ± 0.129	

^†^ 1428 /1674 completed a 12 month visit and completed EQ5D at baseline and 12 months

^‡^ Linear model, dependent variable change in EQ5D, covariates = Age group, baseline, ED5D

^§^ Improvement from baseline to 12 months p = 0.54

^¶^ Improvement from baseline to 12 months p < 0.01

## Discussion

Real-world evidence of the clinical outcomes after CBA in the octogenarian population is sparse. Thus, our study aimed to assess the efficacy and safety of CBA to treat AF in octogenarians, and to the best of our knowledge, this study evaluates the largest cohort of octogenarians treated with CBA, globally. This work is the first real-world analysis of a large global dataset of CBA procedures in octogenarians, including regions with scarce CBA evidence (*i.e.,* Southeast Asia, Middle East, etc.), performed by a vast variety of operators from world-wide healthcare systems. The data indicated high efficacy for CBA in octogenarians, with comparable 12-month freedom from AA recurrence compared to patients < 80 years of age. Even when accounting for differences seen in baseline characteristics and AAD usage by propensity score methods; octogenarians had similar efficacy, safety, and healthcare utilization outcomes in comparison to control. Moreover, both cohorts experienced a decrease in AAD utilization post-ablation and improvement in QoL at 12 months. Of note, these results are based on a high proportion of completed follow-ups of more than 90% of the overall cohort.

These findings are supported by smaller non-global studies. Liu *et al. *reported that 74% of octogenarians who underwent CBA experienced freedom from AA at 12 months [11]. After a one-year follow-up, Abugattas *et al.*and Heeger *et al.*observed 81.1% and 80% of their elder cohort to be in sinus rhythm post CBA (respectively), with no significant difference to controls [12, 13]. However, previous studies evaluating the impact of age on the efficacy of CBA over longer follow-up periods have been less consistent. Vermeersch *et al.*found that after a median follow-up of 24 months post-CBA, patients ≥ 75 years old with PsAF had more AA recurrences compared to PsAF patients < 75 years old (p = 0.03) [14]. Although the freedom from AF was similar between the two groups after a year, the success rate remained consistent in the younger cohort but continued to decline in the older group [14]. Hartl *et al.*found that freedom from arrhythmia was not age dependent at the 12-month follow-up, but it was significantly different between age groups following 36 months post CBA [10]. However, a 2019 CBA study discovered no differences in AF recurrence between ≥ 75 years old patients and the control cohort after three years [13]. Together these findings suggest that more investigation is needed into the durability of CBA in older AF patients.

Overall, it is surprising that (despite the higher rate of comorbidities in the octogenarians) efficacy rates were similar compared to the younger patients. Through propensity score methods, CBA was determined to be just as effective in octogenarians as in the control group despite these differences in cardiovascular health and all other baseline characteristics. Furthermore, this logistic regression model demonstrated that AAD treatment from discharge to 12 months did not contribute to arrythmia recurrence in octogenarians. These results reinforce that CBA is an efficacious treatment for AF in the octogenarian population. It is of note, that there was a trend of a lower rate of repeat ablations performed in the octogenarians, but without full statistical significance. It is likely that physicians were reluctant to perform a second minimally invasive procedure in the elderly and treated them with AADs instead. Bunch *et al. *found that the need for AADs following ablation is more common with increasing patient age [15]. Of note, in our analysis, AF-related symptoms improved as much as in the younger cohort. A recently published randomized trial indicated a reduction of cardiovascular events by early restoration of sinus rhythm in AF patients [16]; however, this is of less relevance for clinical decision making in octogenarians, and instead, indication for rhythm therapy in elder AF patients should be driven by symptoms.

The study data show that CBA was feasible in patients ≥ 80 years of age with similar procedure times and success rates for acute PVI compared to younger patients. Although the elderly suffered from more comorbidities, indicated by higher CHA_2_DS_2_-VASc scores, being more hypertensive, higher NYHA classification, and more frequent prior myocardial infarctions; CBA did not result in a higher rate of serious procedure-related adverse events nor rehospitalizations compared to the control group in this large, global, multicenter registry. Of note, although CHA_2_DS_2_-VASc scores were higher in octogenarians, these patients had a relatively good health status for their age, suggesting that elder patients were selected for PVI based on favorable baseline characteristics and that some physicians were reluctant to perform PVI in more frail patients. In fact, frailty in AF patients is significantly associated with increased symptom severity, incidence of stroke and mortality, and prolonged hospitalization [17, 18]. In this regard, patient selection in the elder population of who may benefit from ablation becomes critical. This should be considered when interpreting the outcomes of this real-life registry.

However, there was a trend for octogenarians to remain in the hospital longer following CBA. This is aligned with previous findings of octogenarians being hospitalized for more than a mean of 2.5 days, and longer hospitalization is often necessary for elder patients in order to enhance fluid levels and improve mobility [15, 19]. Moreover, there may be some fear about delayed complications after PVI in elderly patients, which is why physicians may have been generous in keeping octogenarians in the hospital longer after PVI. Nevertheless, our data indicate that PVI can be safely performed in octogenarians (when selected properly), and thus, discharge does not need to be postponed as a standard practice.

These global results confirm earlier findings from several smaller single center trials [3, 6, 8–10] and multicenter studies in smaller geographies [11–13, 19, 20] in terms of similar complication rates in elder patients despite a higher prevalence of cardiovascular diseases compared to control [3, 6, 8–13, 15, 19–22]. Similar to our results, Abin *et al.*and Hartl *et al.*reported no procedure-related deaths or atrioesophageal fistulas in elder AF patients treated with CBA [9, 10]. However, Hartl *et al.*found that transient ischemic attacks occurred only in the elder group, the opposite result from our study [10]. Even in a study in which two patients from the elder cohort died from cardiovascular complications; there was no significant difference in mortality compared to the control cohort [3]. A 2022 meta-analysis review of 20 studies found that older patients suffered significantly more overall, major, and cerebrovascular complications in comparison to the control group [23]. However, when the investigators analyzed adverse events from solely CBA studies, there were no longer differences in the complication rates between the elder and non-elder cohorts. This observation may be due to the fact that cryoballoon ablation provides procedural standardization, allowing for more consistent clinical outcomes. Furthermore, the most common scenario in which phrenic nerve injury occurs is with CBA, with an incidence of transient phrenic nerve palsy of 3.5% to 11.2% [24–28]; however, no phrenic nerve injuries occurred in the octogenarian cohort. These results support CBA as a safe treatment for octogenarians suffering from AF disease.

It is well established that the incidence of AF increases with age [1]. Surprisingly, octogenarians only made up 2.4% (101/4210) of patients enrolled in the Cryo Global Registry. Excluding Germany, octogenarians across various geographies (Supplemental Table 1) rarely underwent CBA in this large, real-world registry. This novel finding demonstrates that octogenarians are perhaps an underserved AF population, and the results from this study support CBA in older patients.

### Study Limitations

Our study has some limitations. A mandatory, universal protocol for rhythm monitoring was not used across centers. Intermittent monitoring was employed for most patients, and consequentially, AF recurrence may have been under-reported. However, patients in both cohorts were monitored on average three times over 12 months, primarily with Holter and 12-lead ECG monitoring, and there was no statistical difference in the proportion of unmonitored patients between groups. Also, this was a real-world registry accompanied with a selection bias that is common to any observational trial. To circumvent this issue, Cox proportional hazards modeling was utilized to account for differences in baseline characteristics between cohorts. Based on the overall reserved treatment of elders with invasive therapies, only octogenarians of relatively good physical condition may have been treated with CBA and may not reflect the broader population of octogenarians with AF.

## Conclusions

This study establishes CBA as a safe and effective treatment for octogenarians suffering from PAF and PsAF, with efficacy and complication rates comparable to younger patients. Still, when considering older people’s greater risk for comorbidities; great care should be taken when deciding what treatment options for AF to pursue. Therapy options should consider the general health of the patient and life expectancy, the physician’s medical guidance, and the patient’s desire for maintaining physical activity and QoL.

## Supplementary Information

Below is the link to the electronic supplementary material.

Supplementary file1 (DOCX 17 KB)

## Data Availability

The authors confirm that all data generated or analysed during this study are included in this published article.

## Abbreviations

AAD: Antiarrhythmic drug
AA: Atrial arryhtmia
AF: Atrial fibrillation
AFL: Atrial flutter
AT: Atrial tachycardia
CV: Cardiovascular
CBA: Cryoballoon ablation
LA: Left atrium
PAF: Paroxysmal AF
PsAF: Persistent AF
PV: Pulmonary vein
PVI: Pulmonary vein isolation
