# Supplementary material for: Cryoballoon ablation of atrial fibrillation in octogenarians: one year outcomes from the cryo global registry
Source: J Interv Card Electrophysiol. 2023 Dec 12;67(6):1341–51. doi: 10.1007/s10840-023-01680-z (PMC11379788; doi:10.1007/s10840-023-01680-z)
Supplement: Supplementary file 1 — Supplementary file1 (DOCX 17 KB) [file 10840_2023_1680_MOESM1_ESM.docx]

**Supplemental Table 1. Enrollment Distribution by Country**

| **Country** | **Number of Centers** | **Number of**  **Patients** | **Number of Octogenarians** | **Percent Per Country** |
| --- | --- | --- | --- | --- |
| Austria | 1 | 42 | 2 | 5% |
| Belgium | 1 | 27 | 6 | 22% |
| Switzerland | 1 | 19 | 1 | 5% |
| Czech Republic | 1 | 32 | 1 | 3% |
| Germany | 10 | 417 | 43 | 10% |
| Hungary | 2 | 149 | 3 | 2% |
| United Kingdom | 1 | 99 | 2 | 2% |
| China | 2 | 76 | 2 | 3% |
| Taiwan, Republic of | 2 | 59 | 5 | 8% |
| Japan | 6 | 219 | 14 | 6% |
| Korea (South) | 5 | 299 | 12 | 4% |
| Argentina | 1 | 99 | 1 | 1% |
| Malaysia | 1 | 36 | 1 | 3% |
| United States of America | 5 | 101 | 8 | 8% |
| **Total** | **37** | **1674** | **101** | **6%** |
